# Understanding medicine related adverse events in people with multiple long-term conditions and polypharmacy

**DOI:** 10.23889/ijpds.v9i5.2832

**Published:** 2024-09-18

**Authors:** James Rafferty, Jane Lyons, Ronan Lyons, Rhiannon Owen

**Affiliations:** 1 Swansea University Medical School

## Background and Objectives

Polypharmacy is increasingly common in people living with multiple long-term conditions (MLTC), but clinical evidence often does not consider medicine interactions beyond two or three treatments at a time. The aim of this research is to develop statistical models to better understand interactions between treatments leading to adverse events for people living with MLTC.

## Approach

We will develop models of risk of adverse events in people with MLTC and polypharmacy using data in the Secure Anonymised Information Linkage (SAIL) Databank - a trusted research environment with linked electronic health record data at the individual-level for the population of Wales, UK. Taking a flexible Bayesian joint modelling approach, where the posterior risk is the product of several sub-models, will enable the characterisation of this complex system in a way that is interpretable for healthcare decision-makers, leading to improved understanding of risk factors for adverse events.

## Results

The Welsh Multimorbidity e-Cohort (WMC) includes 2.9 million people alive and living in Wales on 1st Jan 2000 with follow-up for 20 years. Individuals in the cohort have 932,552,061 medication items prescribed and 277,279 coded adverse events recorded in primary care data. Modelling approaches will focus on feasibility of fitting models to population-level datasets, and the interpretability of the model results for healthcare decision-making.

## Conclusions and Implications

Results have the potential to inform healthcare policy and practice for the management of people living with MLTC and polypharmacy, thus improving patient outcomes and making the best use of limited healthcare resources.

